# COVID‐19 in patients with Down syndrome: A systematic review

**DOI:** 10.1002/iid3.1219

**Published:** 2024-03-19

**Authors:** Praveen N. K. Pitchan Velammal, Suryakumar Balasubramanian, Fathima Shehnaz Ayoobkhan, Gautham V. K. Mohan, Pearl Aggarwal, Ali A. Rabaan, Syed A. Khan, Farah Yasmin, Thoyaja Koritala, Salim R. Surani

**Affiliations:** ^1^ Department of Medicine Tirunelveli Medical College and Hospital Tirunelveli India; ^2^ Department of Medicine Velammal Medical College Hospital and Research Institute Madurai India; ^3^ Department of Medicine St Vincent Charity Medical Center Cleveland Ohio USA; ^4^ Department of Medicine Johns Hopkins Aramco Healthcare Dhahran Saudi Arabia; ^5^ Department of Medicine Alfaisal University Riyadh Saudi Arabia; ^6^ Department of Medicine The University of Haripur Haripur Pakistan; ^7^ Critical Care Medicine Mayo Clinic Health System Mankato Minnesota USA; ^8^ Department of Medicine Yale University School of Medicine New Haven Connecticut USA; ^9^ Department of Medicine & Pharmacology Texas A&M University College Station Texas USA; ^10^ Department of Medicine & Pharmacology Research Collaborator, Mayo Clinic Rochester Minnesota USA

**Keywords:** clinical outcomes, clinical symptoms, COVID‐19, Down syndrome, mortality

## Abstract

**Introduction:**

Down syndrome (DS) is associated with multiple comorbid conditions and chronic immune dysfunction. Persons with DS who contract COVID‐19 are at high risk for complications and have a poor prognosis. We aimed to study the clinical symptoms, laboratory and biochemical profiles, radiologic findings, treatment, and outcomes of patients with DS and COVID‐19.

**Method:**

We systematically searched PubMed, MEDLINE, Web of Science, Scopus, and the Cochrane Library using the keywords COVID‐19 or coronavirus or SARS‐CoV‐2 and DS or trisomy 21. Seventeen articles were identified: eight case reports and nine case series published from December 2019 through March 2022, with a total of 55 cases.

**Results:**

Patients averaged 24.8 years (26 days to 60 years); 29 of the patients were male. The most common symptoms were fever, dyspnea, and cough. Gastrointestinal and upper respiratory tract symptoms were commonly reported for pediatric patients. The most common comorbidities present in patients with DS were obesity (49.0%), hypothyroidism (21.6%) and obstructive sleep apnea (15.6%). The patients were hospitalized for a mean of 14.8 days. When the patients were compared with the general COVID‐19 population, the mean number of hospitalized days was higher. Most patients had leukopenia, lymphopenia, and elevated inflammatory markers (d‐dimer and C‐reactive protein). Bilateral infiltrations and bilateral ground‐glass opacifications were frequently seen in chest radiographs and chest computed tomographic imaging. Most of the patients were treated with methylprednisolone, macrolides, and hydroxychloroquine. Of the 55 patients, 22 died. The mean age of the patients who died was 42.8 years. Mortality rate was higher in individuals with DS over 40 years of age.

**Conclusion:**

More studies are needed to better understand COVID‐19 infections among persons with DS. In addition, the study was limited by a lack of statistical analyses and a specific comparison group.

## Graphical abstract

The present study is based on a systematic review of cases of patients with Down syndrome and COVID‐19.

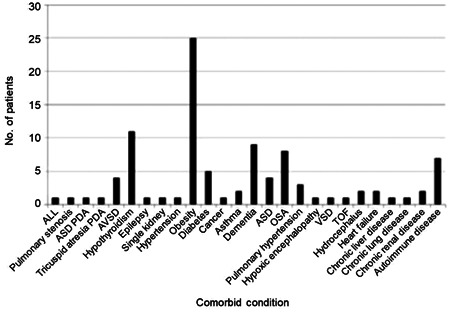

## 1 INTRODUCTION

COVID‐19 has affected approximately 522 million people, and over six million people have died globally since the onset of the pandemic.^1^ Patients with comorbid conditions have worse outcomes and higher mortality rates.^2^, ^3^ Diabetes, hypertension, and coronary artery disease are the major risk factors for the severity of COVID‐19,^4^ and individuals with intellectual disabilities continue to be among the most vulnerable members of society.^5^

Down syndrome (DS) is the most common genetic cause of mental impairment in the world, affecting anywhere from 1 in 400 to 1 in 1500 people depending on the population studied, maternal age, and prenatal screening schedules.^6^ The most common associated anomalies are cardiac, followed by digestive system anomalies, musculoskeletal system anomalies, urinary system anomalies, and respiratory system anomalies. Among these disorders, congenital heart diseases are most prevalent.^7^ Adults with DS are more likely to have overweight or obesity than other adults.^8^ Obesity in persons with DS is associated with obstructive sleep apnea, dyslipidemia, hyperinsulinemia, and gait disorder.^8^ DS also causes a state of chronic immune dysfunction, and persons with DS are more prone to respiratory tract infections. Those with congenital heart anomalies or those who have bronchopneumonia are more likely to die than persons in the general population.^9^ Because of these issues, persons with DS are more susceptible to COVID‐19. In addition, persons with DS depend on support from various social services and other people,^10^ exposing them to more possibilities of infection. According to the Trisomy 21 Research Surgery, 1046 patients with DS were infected with COVID‐19 between April 2020 and October 2020, and 422 were hospitalized. When data from this study was compared with that of the UK ISARIC4C survey, a significant increase in morality was observed among persons with DS, particularly after the age 40.^11^

Anti‐inflammatory, antibiotics, antivirals, and immunomodulators played a vital role in the treatment of the patients with DS, reducing the impact of the infections. Limited clinical and research studies have been published describing the effect of COVID‐19 on persons with DS. To learn more about the effect of COVID‐19 on persons with DS, we systematically reviewed the literature for reports of COVID‐19 in patients with DS, focusing on clinical symptoms, laboratory and biochemical profiles, radiologic findings, treatment, and outcome. In this article, we focus mainly on the clinical manifestations, treatment, and outcome of DS. This study opens up the possibility of understanding the manifestations and consequences of COVID‐19 infections in DS patients, as well as formulating treatment protocols.

## 2 METHODS

We followed the Preferred Reporting Items for Systematic Reviews and Meta‐analyses guidelines for this study.

### 2.1 Search strategy and data sources

To find studies reporting the clinical features of COVID‐19 among patients with DS, a systematic search was conducted in PubMed, MEDLINE, Web of Science, Scopus, and the Cochrane Library from December 1, 2019, through March 31, 2022, using the key terms COVID‐19 or coronavirus or SARS‐CoV‐2 and DS or trisomy 21. We applied no restrictions based on language, geographical location, and year of publication. We reviewed the reference lists of the papers we identified to find additional reports.

### 2.2 Study selection

If the abstract provided insufficient information to determine eligibility, the paper was retrieved for review Only manuscripts with clinical, biochemical, radiologic, therapeutic, and outcome data on DS and COVID‐19 were considered. If the full‐text paper was not accessible, original data were not given (e.g., review articles), or other coronavirus serotypes were identified as the infective agents, the manuscripts were excluded from review. We only included studies published in the English language in our systematic review.

### 2.3 Data extraction

Data identification and extraction from publications were standardized using a structured data extraction form. Published biochemical and laboratory data that were presented as absolute values were compared with globally known ranges to assess normalcy.^12^ Parameters were reported as high, normal, or low, with no values given.

## 3 RESULTS

We retrieved 400 articles from the search, of which 17 were determined to be appropriate for data extraction. There were 55 cases described in the 17 articles (eight case reports and nine case series).

### 3.1 Demographic characteristics

**Figure 1:**
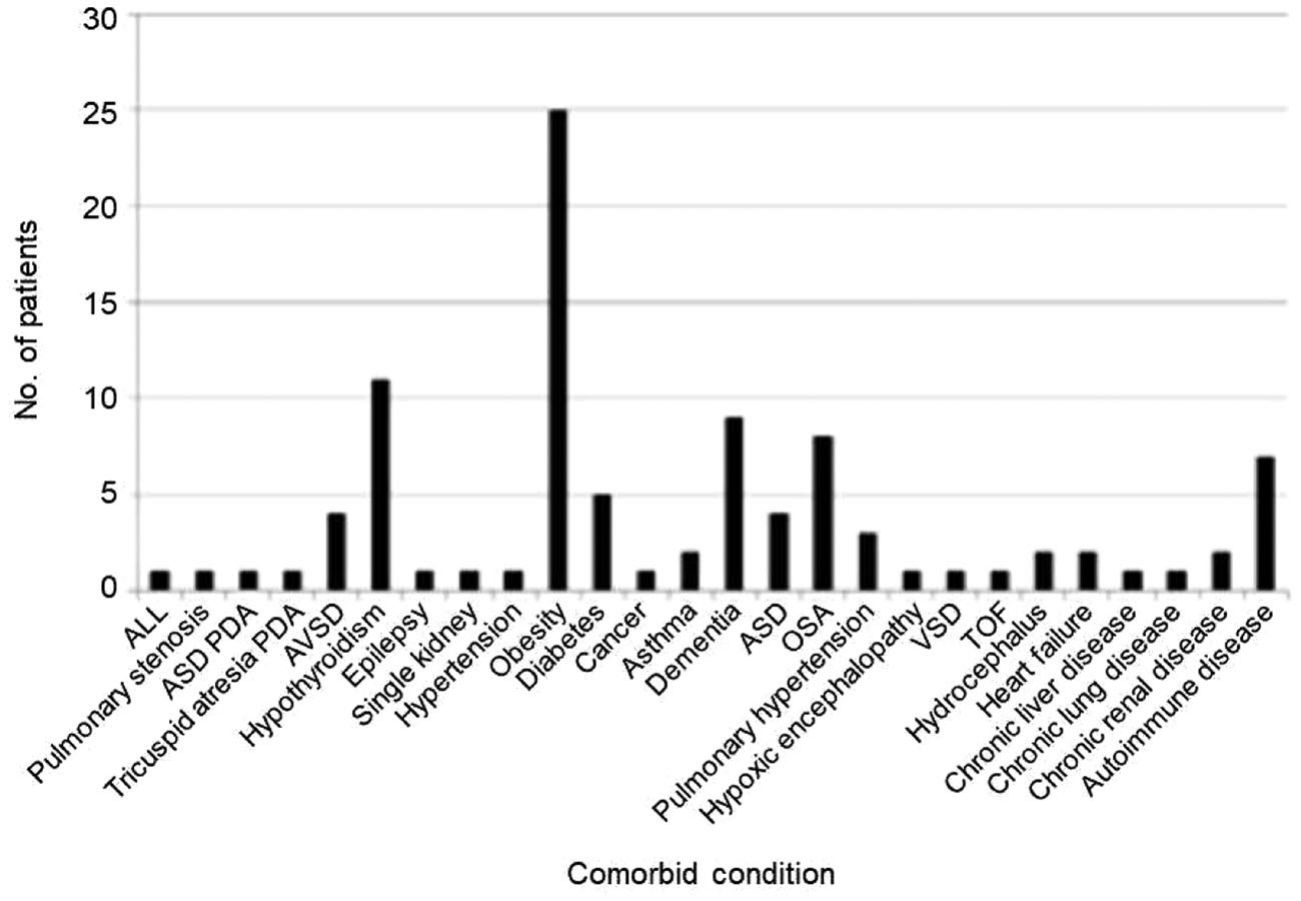
Comorbid conditions of patients with COVID‐19 and Down syndrome. ALL, acute lymphoblastic leukemia; ASD, atrial septal defect; AVSD, atrioventricular septal defect; OSA, obstructive sleep apnea; PDA, patent ductus arteriosus; TOF, tetralogy of Fallot; VSD, ventricular septal defect.

The mean age of the patients was 24.8 years (26 days to 60 years), and 29 of the 55 patients were male (Supporting Information S1: Table 1).^13^, ^14^, ^15^, ^16^, ^17^, ^18^, ^19^, ^20^, ^21^, ^22^, ^23^, ^24^, ^25^, ^26^, ^27^, ^28^ Comorbid conditions were described for 51 cases. Obesity was the most common comorbid condition reported (49.0%, 25) followed by hypothyroidism (21.6%, 11) (Figure 1, Supporting Information S1: Table 1).

### 3.2 Symptoms

The following symptoms were reported by patients 18 years and older (*n *= 29): fever, 79.3% (23); dyspnea, 65.5% (19); cough, 31% (nine); dysphagia, 10.3% (three); hemoptysis, 6.9% (two); myalgia, 6.9% (two); diarrhea, 3.4% (one); nasal congestion, 3.4% (one); fatigue, 3.4% (one); vomiting, 3.4% (one); decreased urine output, 3.4% (one); and stupor, 3.4% (one) (Supporting Information S1: Table 1).

The following symptoms were reported by patients younger than 18 years (*n* = 14): fever, 100% (14); cough, 57.1% (eight); dyspnea, 57.1% (eight); nasal congestion, 35.7% (five); diarrhea, 35.7% (five); fatigue, 21.4% (three); sore throat, 14.3% (two); vomiting, 21.4% (three); sneezing, 7.1% (one); cyanosis, 14.3% (two); abdominal pain, 14.3% (two); dysphagia, 7.1% (one); altered sensorium, 7.1% (one); rash, 7.1% (one); decreased urine output, 7.1% (one); and weight loss, 7.1% (one) (Supporting Information S1: Table 1).

### 3.3 Hospitalization and level of care

Most patients began care in the intensive care unit (ICU) (63.6%, 35/55), where they received respiratory support (*n* = 35): oxygen therapy, 28.5% (10); noninvasive ventilator support, 14.2% (five); mechanical ventilator support, 48.5% (17); and high‐flow nasal cannula, 5.7% (two) (Supporting Information S1: Table 2). The mean hospitalization was 48 days (Supporting Information S1: Table 2).

### 3.4 Laboratory and radiologic findings

**Figure 2:**
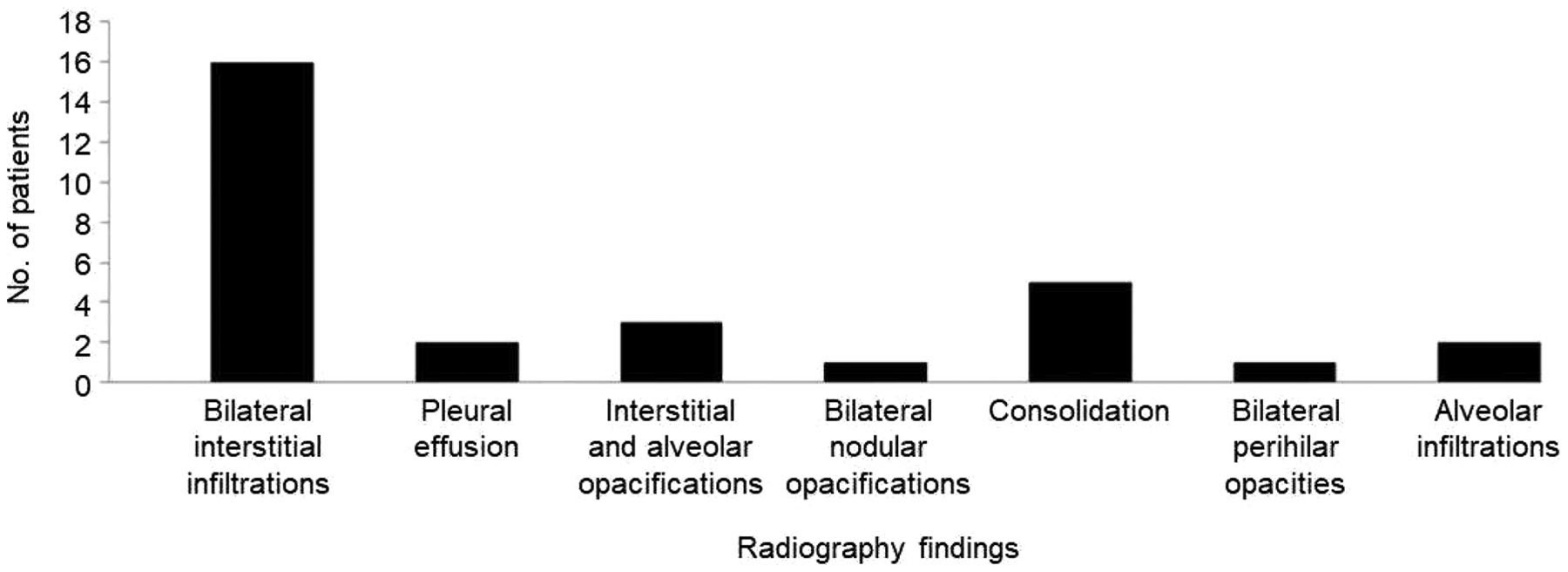
Radiographic findings of patients with COVID‐19 and Down syndrome (*n* = 16).

**Figure 3:**
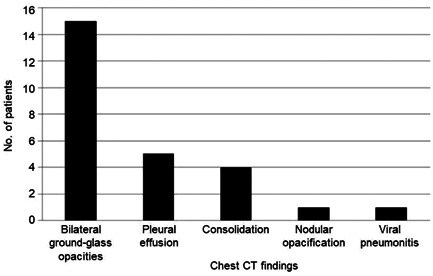
Findings from chest computed tomography (CT) for patients with COVID‐19 and Down syndrome.

The articles included a wide range of laboratory and radiologic results. For 54 patients, COVID‐19 was diagnosed by a positive polymerase chain reaction test for SARS‐CoV‐2, and one patient's case was diagnosed by serologic testing for SARS‐CoV‐2 (immunoglobulin [Ig] M and IgG) (Supporting Information S1: Table 3). Most patients had elevated levels of the inflammatory markers C‐reactive protein (94.3%, 50/53) and d‐dimer (100%, 30/30) as well as leukopenia (92.0%, 46/50) and lymphopenia (89.6% 43/48) (Supporting Information S1: Table 3). There were radiologic findings in 60.0% of cases (33/55). Most patients had chest radiography (75.8%, 25/33) (Supporting Information S1: Table 4). Findings from the radiographs (*n* = 25) were mainly bilateral infiltrations (64.0%, 16) (Supporting Information S1: Table 4, Figure 2). About half of the patients (54.5%) had chest computed tomography (18/33) (Supporting Information S1: Table 4, Figure 3). Reported chest computed tomography findings (*n* = 18) were mainly bilateral ground‐glass opacifications (88.8%, 16), pleural effusion (27.7%, five), and consolidation (22.2%, four).

### 3.5 Treatment and outcomes

**Figure 4:**
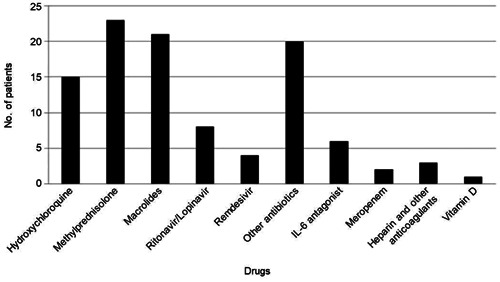
Drugs used to treat patients with covid‐19 and Down syndrome (*n* = 28 patients). IL‐6, interleukin 6.

Patients were treated with various anti‐inflammatory drugs and antibiotics (*n* = 40) including methylprednisolone (57.5%, 23), macrolides (52.5%, 21), other antibiotics (50.0%, 20), hydroxychloroquine (37.5%, 15), ritonavir/lopinavir (20.0%, eight), interleukin (IL)‐6 antagonists (sarilumab/tocilizumab) (15.0%, six), and remdesivir (10.0%, four) (Supporting Information S1: Table 5, Figure 4). The reported mortality was 49.0% (27/55); however, the causes of death were not reported; we could only learn whether the patients recovered or died (Supporting Information S1: Table 5). The mean age of patients who died was 42.8 years.

## 4 DISCUSSION

### 4.1 Symptoms and comorbid conditions

The reported symptoms (fever, 86%; dyspnea, 62.7%; and cough, 37.2%) were similar to symptoms described for the general population by Grant et al.^29^ For children (younger than 18 years), gastrointestinal and upper respiratory symptoms were most common, which are similar to findings in the general pediatric COVID‐19 population studies.^30^, ^31^ In this study, 49.0% of patients had obesity, and 15.6% had obstructive sleep apnea (15.6%, eight). For patients with COVID‐19, obesity is a major risk factor for increased ICU admission and death.^32^ Patients with DS often have associated obesity and obstructive sleep apnea, both of which could compromise the outcome. Indirectly, obstructive sleep apnea could make COVID‐19 more severe by increasing hypoxemia and pulmonary hypertension and decreasing cardiopulmonary capacity.^33^

### 4.2 Hospitalization and level of care

The mean number of hospitalized days among patients with DS was 14.8 days, which is similar to findings of a study by Hüls et al.^11^ and Malle et al.^27^ that reported on patients with DS and COVID‐19. The mean number of hospitalized days was greater (median, 16 days) compared with hospitalized days in the general population of patients with COVID‐19 (median, 7 days).^27^, ^34^ Most patients were admitted to the ICU (63.6%) and underwent respiratory support, which is similar to findings of another report.^35^

### 4.3 Laboratory investigations

Patients in this study had elevated levels of C‐reactive protein (94.3%) and d‐dimer (100%) as well as leukopenia (92.0%) and lymphopenia (96.2%). DS is associated with immune dysregulation,^36^ and trisomy 21 cells overproduce the pro‐inflammatory cytokines and IL‐10. Patients with DS are, therefore, more vulnerable to cytokine storm and increased mortality because of this overproduction of pro‐inflammatory cytokines. IL‐10 overproduction suppresses the antibacterial effects of the immune system, which increases the risk of superinfections in patients with DS.^35^

### 4.4 Cytokine storm

In persons with DS, there is triplication of four interferon (IFN) receptors: the two type I IFN receptors (*IFNAR1* and *IFNAR2*), the type II IFN receptor (*IFNGR2)*, and *IL10RB*, which is a receptor subunit for both type III ligands as well as cytokines IL‐10, IL‐22, and IL‐26.^33^ Overexpression of IFN receptors causes hypersensitivity to IFN type I and IFN type III when epithelial cells in the lungs are exposed to SARS‐CoV‐2.^33^ In persons with DS, the primary immune cell types are dendritic cells and T cells. These cells display changes indicative of hyperactivation and increased differentiation in inflammatory states.

This heightened immune activity could predispose individuals with DS to cytokine overproduction and an increased risk of acute respiratory distress syndrome, myocardial damage, organ failure, and secondary bacterial infections.

Hence, this situation supports the notion that individuals with DS are more likely to develop cytokine release syndrome or hypercytokinemia,^37^, ^38^ and supports the use of combinations of antiviral treatments and targeted immunosuppression in the treatment of persons with DS and COVID‐19.^33^, ^39^, ^40^, ^41^

### 4.5 Radiologic findings

The most common radiographic findings reported for patients in this study were bilateral infiltration (64.0%), and the most common computed tomography findings were bilateral ground‐glass opacifications (88.8%), findings similar to those reported by Kaufman et al. in a general population study.^42^ The most common radiographic findings were bilateral infiltration (64%), and the most common computed tomographic findings were bilateral ground‐glass opacifications (89%), findings that are similar to those reported in a general population study.^42^

### 4.6 Treatment and outcomes

Patients with DS and COVID‐19 are commonly treated with the following drugs: methylprednisolone (57.5%), macrolides (52.5%), and hydroxychloroquine (37.5%), which were similar to treatments used for the general population.^43^, ^44^

### 4.7 Multisystem inflammatory syndrome in children and COVID‐19

A case of multisystem inflammatory syndrome in a child with DS and COVID‐19 has been reported,^15^ and the case was reviewed for this study. The patient, whose COVID‐19 diagnosis was made through serologic findings (IgM, IgG), had a Kawasaki‐like illness, which was similar to the type of illness reported for children with the disorder in the general pediatric population.^45^ The patient was treated with intravenous immunoglobulins and aspirin.

### 4.8 Age and outcomes

Patient age is closely linked to hospitalization rates and mortality for patients with DS and COVID‐19. In the current study, the mortality rate increased at age 40 years, which is much younger than the increase in mortality rate for the general population.^27^ Persons with DS have a lifespan of about 20 years less (between 55 and 60 years) than that of the general population, which likely accounts for the age difference in outcomes.^24^ The Trisomy 21 Research Society conducted a large survey of persons with DS who had COVID‐19 or had symptoms of COVID‐19 (*N* = 1046), and most persons were symptomatic (*N* = 981). Of 861 who were tested for COVID‐19, 750 were positive. As in this study, an increase in fatal outcomes occurred at age 40, about 10 years earlier than in the general population.^46^

### 4.9 COVID‐19 vaccinations and DS

Considering the high risk of severe disease, morbidity, and mortality, COVID‐19 vaccines were initially prioritized for high‐risk groups.^47^ In December 2020, the Centers for Disease Control and Prevention added persons with DS to high‐risk categories.^48^ According to the study conducted by Hüls et al. individuals with DS were vaccinated against COVID‐19. It was found to be safe and effective in patients with DS. Following their COVID‐19 vaccination, more than half of the DS patients (first dose: 1048 [53%], second dose: 962 [56%]) reported no side effects. Acute injection site pain (first dose: 578 (29% of those who received a first dose, second dose: 492 (29% of those who received a second dose), fatigue (first dose): 217 (11%), second dose: 235 (14%) and fever (first dose): 183 (9%), second dose: 90 (5%) were the most common side effects identified.^49^

### 4.10 Limitations

More studies are needed to better understand COVID‐19 infections among persons with DS. In addition, the review was limited by a lack of statistical analyses, quantitave synthesis, and a specific comparison group. Finally, we were only able to include case reports, and case series in our systematic review due to lack of randomized control trials and observational studies in published literature. This raises the possibility of a potential publication bias in our findings.

## 5 CONCLUSION

The present study is based on a systematic review of cases of patients with DS and COVID‐19. The clinical symptoms, biochemical findings, laboratory findings, radiologic findings, and treatments were similar to those same parameters in the general population of patients with COVID‐19. However, persons with DS who died of COVID‐19 were younger and had different comorbid conditions than those without DS. The comorbidity burden can influence COVID‐19 prognosis in persons with DS. A focus should therefore be placed on preventing COVID‐19 as well as on treating patients with DS earlier and more aggressively because they have a higher risk of hospitalization and mortality from the disease.

## AUTHOR CONTRIBUTIONS

**Praveen N. K. Pitchan Velammal**: Conceptualization; data curation; formal analysis; investigation; project administration; methodology; resources; validation; visualization; writing original draft. **Suryakumar Balasubramanian**: Formal analysis; validation; visualization; writing review and editing. **Fathima Shehnaz Ayoobkhan**: software. **Gautham V. K. Mohan**: software. **Pearl Aggarwal**: Software. **Syed A. Khan**: Writing review and editing. **Salim R. Surani** Supervision; writing review and editing; validation. **Thoyaja Koritala**: Supervision; writing review and editing; validation. **Ali A. Rabaan**: Writing review and editing. **Farah Yasmin**: Supervision, writing review and editing, validation.

## CONFLICT OF INTEREST STATEMENT

The authors declare no conflict of interest.

## Supporting information

Supporting information.

Supporting information.

## Data Availability

The data that supports the findings of this study are available within the article and its Supporting Information material.
