# Supplementary material for: COVID‐19 in patients with Down syndrome: A systematic review
Source: Immun Inflamm Dis. 2024 Mar 19;12(3):e1219. doi: 10.1002/iid3.1219 (PMC10949394; doi:10.1002/iid3.1219)
Supplement: Supplementary file 2 — Supporting information. [file IID3-12-e1219-s002.docx]

**Supplemental Tables**

COVID-19 positive Down syndrome cases.

**Supplemental Table 1.** Clinical Symptoms and Comorbidities Among Patients With COVID-19 and Down Syndrome

| **Study No** | **Author** | **Country** | **No of patients** | **Age** | **Sex** | **Clinical symptoms** | **Co-morbidities** |
| --- | --- | --- | --- | --- | --- | --- | --- |
| 1 | Pontes et al [13] | Brazil | 2 | P1-26  days | M | Nasal congestion,  fever, dyspnea | Atrial Septal Defect (ASD) |
|  |  |  |  | P2-9  mos |  | Fever, nasal  congestion, dyspnea | NA |
| 2 | De  Cauwer et al [14] | Belgium | 3 | P1-60  yrs | F | Fever, cough, stupor | NA |
|  |  |  |  | P2-48  yrs | F | Fever, cough, dyspnea |  |
|  |  |  |  | P3-55  yrs | F | Fever, dyspnea |  |
| 3 | Kantar et al [15] | Italy | 2 | P1-34  months | F | Kawasaki like illness (MIS-C) | ASD |
|  |  |  |  | P2-14  yrs | F | Fever, cough, nasal congestion, sore throat, dyspnea,  fatigue | Obesity, Obstructive Sleep Apnea (OSA) |
| 4 | Krishna n et al [16] | USA | 3 | P1-3  yrs | M | Fever, cyanosis, lethargy | Atrio-Ventricular septal Defect (AVSD), Pulmonary Hypertension, OSA, hypoxic  encephalopathy |
|  |  |  |  | P2-25  yrs | F | Fever, vomiting, decrease urine output,  lethargy | AVSD with  Eisenmenger syndrome, OSA |
|  |  |  |  | P3-21  yrs | M | Fever, cough, dyspnea | Obesity, AVSD, OSA, Pulmonary  hypertension |
| 5 | Newma n et al [17] | USA | 4 | P1-17  yrs | M | Fever, sore throat,  cough, difficulty while eating | Obesity, Ventricular  Septal Defect (VSD), OSA |
|  |  |  |  | P2-10  months | M | Fever, cough, dyspnea | Obesity, Tetrology of Fallot, OSA, Pulmonary  hypertension |
|  |  |  |  | P3-15  yrs | M | Fever, vomiting, cough | Obesity, OSA, ASD,  Hypothyroidism, epilepsy |
|  |  |  |  | P4-  14yrs | M | Fever, abdominal pain, vomiting,  cough, fatigue | Obesity, OSA, ASD |

| 6 | Vita et al [18] | Italy | 2 | P1- 59  yrs | F | Fever, dyspnea | Congenital  hydrocephalus, epilepsy, hypothyroidism |
| --- | --- | --- | --- | --- | --- | --- | --- |
|  |  |  |  | P2- 42  yrs | F | Fever | Hypothyroidism |
| 7 | Villani et al [19] | Italy | 16 | 52.3±7.  3 | F=6  (37.  5%) | Fever (12/16), dyspnea (11/16), cough (3/16), diarrhoea (1/16), hemoptysis (2/16) | Heart Failure- (2/16), Dementia- (6/16), Chronic liver disease- (1/16),  Chronic renal disease- (2/16), Chronic lung disease- (1/16), Autoimmune- (7/16), Obesity-  (6/16) |
| 8 | Arafat et al [20] | Russia | 1 | 6 yrs | M | Fever | Acute Lymphoblastic Leukemia (ALL) |
| 9 | Malik & Kathuria  [21] | India | 1 | 12  months | M | Fever, altered  sensorium, dyspnea, diarrhea | Pulmonary Stenosis (PS) |
| 10 | Stefanut o et al [22] | Brazil | 1 | 9 yrs | F | Fever, cough, dyspnea, weight loss | ASD with Patent ductus arteriosus (PDA) |
| 11 | Vazquez- Hernánde z et al [23] | Mexico | 1 | 2 yrs | F | Abdominal pain, diarrhea, dyspnea, fever, cyanosis, oliguria | Tricuspid atresia, Pulmonary Stenosis (PS) |
| 12 | Alsahabi et al [24] | Saud i Arab  ia | 1 | 4  months | F | Fever, dyspnea, diarrhea, vomiting, cough | Unrepaired Atrioventricular Septal Defect (AVSD) |
| 14 | El  Kaouini et al [25] | Moro cco | 2 | P1- 27  yrs | M | Dyspnea, fever, myalgia, asthenia | Hypothyroidism |
|  |  |  |  | P2- 49  yrs | M | Dyspnea, myalgia, anemia | Epilepsy,  Congenital Hydrocephalus |
| 15 | Uggeri et al [26] | Italy | 1 | 40 yrs | F | Fever, cough, pharyngody  nia | Solitary Kidney |
| 16 | Malle et al [27] | USA | 12 | Mean  age-54 yrs | M-6  F-6 | NA | Hypertension-1, obesity- 12, diabtes-5, cancer-1, asthma-1,  epilepsy-3, dementia-3, hypothyroid-6 |
| 17 | Silva et al [28] | Brazil | 3 | P1- 47  yrs | F | Odynophagia, fever,  cough, dyspnea | NIL |
|  |  |  |  | P2- 33  yrs | F | Dyspnea, fever, cough,  diarrhea, odynophagia | Obese, hypothyroid |

|  |  |  |  | P3- 29  yrs | M | Asthma, rhinitis | Asthma, past H/0 Cardiac surgery |
| --- | --- | --- | --- | --- | --- | --- | --- |
| Abbreviation: P, patient. | | | | | | | |

Kumar Pitchan Velammal et al -24- (MM)

**Supplemental Table 2.** Type of Respiratory Support Used for Patients With COVID-19 and Down Syndrome

| **Name of the author** | **Total Number of**  **patients** | **ICU**  **admission** | **Oxygen therapy** | **NI V** | **MV** | **HFN C** | **No of days admitted in the hospital** |
| --- | --- | --- | --- | --- | --- | --- | --- |
| Pontes et al [13] | 2 | 1 | - | - | - | - | 17 days |
|  |  | 1 | 1 | - | - | - | 17 days |
| De Cauwer et al [14] | 3 | 3 | 3 | - | - | - | - |
| Kantar et al [15] | 2 | 1 | 1 | 1 | - | - | P1-15 days P2- 14 days |
| Krishnan et al [16] | 3 | 3 | - | 3 | - | - | - |
| Newman et al [17] | 4 | 4 | 1 | 1 | 1 | 1 | P1-23  daysP2  -7  daysP3  -4  daysP4  - 2days |
| Vita et al [18] | 2 | 2 | 1 | - | 1 | - | P1-13  daysP2- 29days |
| Villani et al [19] | 16 | 4 | - | - | 4 | - | - |
| Arafat et al [20] | 1 | 0 | - | - | - | - | 26 days |
| Malik & Kathuria [21] | 1 | 1 | - | - | 1 | - | 3 days |
| Stefanuto et al [22] | 1 | 1 | 1 | - | - | - | 10 days |
| Vazquez-Hernandez  et al [23] | 1 | 1 | - | - | 1 | - | 24 days |
| Alsahabi et al [24] | 1 | 1 | 1 | - | - | - | 10 days |
| El Kaouini et al [25] | 2 | 2 | 1 | - | 1 | - | P1- 8 days |
|  |  |  |  |  |  |  | P2- 11 days |
| Uggeri et al [26] | 1 | 1 | - | - | - | 1 | 21 days |
| Malle et al [27] | 12 | 6 | - | - | 5 | - | 15.8 days |
| Silva et al [28] | 3 | 3 | - | - | 3 | - | 13 days |
| Mean hospitalized days: 14.8 | | | | | | | |

Kumar Pitchan Velammal et al -25- (MM)

**Supplemental Table 3.** Laboratory Evaluations for Patients With COVID-19 and Down Syndrome

| **Name of the author** | | **Confirmed cases** | **WBC**  **10³/µL N=3.5-11** | **Lymphocytes**  **10t/μL**  **N=1-4.8** | **CRP**  **mg/L N<5** | **d- dimer μg/L N 0-**  **549** |
| --- | --- | --- | --- | --- | --- | --- |
| Pontes et al [13] | P1 | SARS-CoV-2 PCR  + | leukopeni a | lymphopenia | High | High |
|  | P1 | SARS-CoV-2 PCR + | leukopenia | lymphopenia | High | High |
| De Cauwer et al [14] | P1 | SARS-CoV-2  PCR + | leukopeni  a | Normal | High | High |
|  | P2 | SARS-CoV-2  PCR + | leukopeni  a | lymphopenia | High | High |
|  | P3 | SARS-CoV-2  PCR + | leukopeni  a | lymphopenia | High | High |
| Kantar et al [15] | P1 | Serology for SARS-CoV-2 (IgM, IgG) +  (MIS-C) | leukopeni a | lymphopenia | High | - |
|  | P2 | SARS-CoV- 2 PCR  + | leukopeni a | lymphopenia | High | - |
| Krishnan et al [16] | P1 | SARS-CoV-2 PCR  + | leukopeni  a | lymphopenia | High | High |
|  | P2 | SARS-CoV-2 PCR  + | leukopeni a | lymphopenia | High | High |
|  | P3 | SARS-CoV-2 PCR  + | leukopeni  a | lymphopenia | High | High |
| Newman et al [17] | P1 | SARS-CoV-2  PCR + | leukopeni  a | - | Normal | High |
|  | P2 | SARS-CoV-2  PCR + | Leukocyt  o-sis | - | Normal | - |
|  | P3 | SARS-CoV-2  PCR + | leukopeni a | - | High | High |
|  | P4 | SARS-CoV-2  PCR + | leukopeni a | - | Normal | - |
| Vita et al [18] | P1 | SARS-CoV-2 PCR+ | NA | lymphopenia | High | High |
|  | P2 | SARS-CoV-2 PCR+ | NA | lymphopenia | High | High |
| Villani et al [19] | 16  case s | SARS-CoV-2 PCR+ | leukopeni a | lymphopenia | High | High |
| Arafat et al [20] | P1 | SARS-CoV-2 PCR + | Normal | lymphopenia | High | NA |
| Malik & Kathuria [21] | P1 | SARS-CoV-2 PCR + | leukopenia | lymphopenia | High | NA |

Kumar Pitchan Velammal et al -26- (MM)

| Stefanuto et al [22] | P1 | SARS-CoV-2 PCR + | NA | NA | NA | NA |
| --- | --- | --- | --- | --- | --- | --- |
| Vazquez-Hernandez et al [23] | P1 | SARS-CoV-2 PCR + | Normal | NA | NA | NA |
| Alsahabi et al [24] | P1 | SARS-CoV-2 PCR + | Normal | Normal | High | NA |
| El Kaouini et al [25] | P1 | SARS-CoV-2 PCR + | NA | lymphopenia | High | High |
|  | P2 | SARS-CoV-2 PCR + | NA | lymphopenia | High | NA |
| Uggeri et al [26] | P1 | SARS-CoV-2 PCR + | leukopenia | NA | High | NA |
| Malle et al [27] | 12  cases | SARS-CoV-2 PCR + | leukopenia | lymphopenia | High | NA |
| Silva et al [28] | P1 | SARS-CoV-2 PCR + | leukopenia | lymphopenia | High | NA |
|  | P2 | SARS-CoV-2 PCR + | leukopenia | lymphopenia | High | NA |
|  | P3 | SARS-CoV-2 PCR + | leukopenia | lymphopenia | High | NA |
| Abbreviation: P, patient. | | | | | | |

Kumar Pitchan Velammal et al -27- (MM)

**Supplemental Table 4.** Chest Radiography and Computed Tomography Findings of Patients With COVID-19 and Down Syndrome

| **Name of the author** | **No. of cases** | **Radiological Findings** |
| --- | --- | --- |
| Pontes et al [13] | P1 | Chest Xray- interstitial and alveolar opacities  Chest CT- diffuse ground glass opacification, |
|  | P1 | Chest Xray- mixed interstitial and alveolar opacities with right lung |
| De Cauwer et al [14] | P1 | Chest CT- bilateral ground-glass opacity and  consolidation |
|  | P2 | Chest CT- viral pneumonitis |
|  | P3 | Chest CT- bilateral ground-glass opacity and  consolidation |
| Kantar et al [15] | P1 | Chest Xray- bilateral interstitial pneumonia |
|  | P2 | Chest Xray- bilateral interstitial pneumonia Chest CT-diffuse ground glass opacities |
| Krishnan et al [16] | P1 | Chest Xray- Consolidation, Pneumonia  Chest CT-Consolidation, Pneumonia |
|  | P2 | Chest Xray- bilateral infiltrates |
|  | P3 | Chest Xray- Consolidation, Pneumonia |
| Newman et al [17] | P1 | Chest Xray-bilateral nodular opacities |
|  | P2 | Chest Xray- bilateral perihilar opacities |
|  | P3 | Chest Xray- consolidation |
|  | P4 | Chest Xray- Interstitial and Alveolar Opacifications |
| Vita et al [18] | 2 cases | NA |
|  |  | NA |
| Villani et al [19] | 16 cases | Chest Xray(6/16): Bilateral infiltration- 6(100%),Pleural effusuion-1(16.7%) Chest CT(8/16): Bilateral ground-glass opacification-8 (100%)  Nodules-1 (12.5%)  Pleural effusions-5 (62.5%) |
| Arafat et al [20] | P1 | Chest CT- Patchy nodular consolidations, peripheral ground-glass opacities of both lungs and bilateral pneumonia |
| Malik & Kathuria et al [21] | P1 | Chest Xray- No Significant |
| Stefanuto et al [22] | P1 | Chest Xray-Diffuse alveolar infiltration  Chest CT-multiple confluent consolidations |
| Vazquez- Hernandez et al [23] | P1 | Chest Xray-presence of diffuse alveolar infiltrates in both lungs |
| Alsahabi et al [24] | P1 | Chest Xray- No Significant |
| El Kaouini et al [25] | P1 | Chest CT- bilateral ground glass pneumonia |
|  | P2 | Chest CT- bilateral ground glass pneumonia |

Kumar Pitchan Velammal et al -28- (MM)

| Uggeri et al [26] | P1 | Chest Xray- bilateral alveolar consolidation in both lungs |
| --- | --- | --- |
| Malle et al [27] | 12 cases | NA |
| Silva et al [28] | P1 | Chest Xray- Bilateral interstitial infiltrate with  consolidation |
|  | P2 | NA |
|  | P3 | NA |
| Abbreviation: P, patient. | | |

Kumar Pitchan Velammal et al -29- (MM)

**Supplemental Table 5.** Drugs Used to Treat COVID-19 and Outcomes for Patients With Down Syndrome

| **Name of the author** | **Cases** | **Age** | **Treatment** | **Outcome** |
| --- | --- | --- | --- | --- |
| Pontes et al [13] | P1 | 26 days old-  M | Clarithromycin, other antibiotics | RECOVERED |
|  | P1 | 9 months-M | clarithromycin | RECOVERED |
| De Cauwer et al [14] | P1 | 60 yrs-F | Meropenem | RECOVERED |
|  | P2 | 48 yrs-F | Chloroquine, azithromycin, other  antibiotics | RECOVERED |
|  | P3 | 55 yrs-F | Chloroquine, azithromycin, other  antibiotics | EXPIRED |
| Kantar et al [15] | P1 | 34 months old-F | azithromycin  intravenous immunoglobulin (IVIG), methylprednisone | RECOVERED |
|  | P2 | 14 Yrs-F | azithromycin,  antiviral drugs (lopinavir and ritonavir),  hydroxychloroquine, other antibiotics | RECOVERED |
| Krishnan et al [16] | P1 | 5 yrs-M | methylprednisone, azithromycin, and  hydroxychloroquine | RECOVERED |
|  | P2 | 25 yrs-F | Hydroxychloroquine, azithromycin,  sarilumab, an IL-6 receptor antagonist | RECOVERED |
|  | P3 | 21 yrs-M | Azithromycin, hydroxychloroquine  Tocilizumab | RECOVERED |
| Newman et al [17] | P1 | 17 yrs-M | Hydroxychloroquine remdesivir | RECOVERED |
|  | P2 | 10 months-  M | NA | RECOVERED |
|  | P3 | 15 yrs-M | NA | RECOVERED |
|  | P4 | 14 yrs-M | NA | RECOVERED |
|  | P1 | 59 yrs-F | Methylprednisolone, Lopinavir/ritonavir, other antibiotics | EXPIRED |

Kumar Pitchan Velammal et al -30- (MM)

| Vita et al [18] | P2 | 42 yrs-F | Methylprednisolone, Hydroxychloroquine, liponavir/ritonavir, sarilumab, other antibiotics | RECOVERED |
| --- | --- | --- | --- | --- |
| Villani et al [19] | 16 cases | 52.3 ± 7.3 | Antibiotics- 13 (81.3%), Antivirals-5  (31.25%), hydroxychloroquine-5  (31.25%), Steroids-12 (75%),  Tocilizumab-1 (6%) | EXPIRED-16 |
| Arafat et al [20] | P1 | 6 years 6 months-M | Hydroxychloroquine, Azithromycin, Meropenum, Tocilizumab | RECOVERED |
| Malik & Kathuria et al [21] | P1 | 1 year-M | Remdesivir, Dexamethasone and Heparin and other anti-coagulants | EXPIRED |
| Stefanuto et al [22] | P1 | 9 years-F | Vitamin D and Corticosteroids | RECOVERED |
| Vazquez- Hernandez et al [23] | P1 | 2 years-F | Oseltamivir, Ceftriaxone, Oxacillin,  Azithromycin | EXPIRED |
| Alsahabi et al [24] | P1 | 4 months-F | cefuxoime | RECOVERED |
| El Kaouini et al [25] | P1 | 27 years-M | Piperacillin-tazobactam, Dexamethasone and Enoxaparin | RECOVERED |
|  | P2 | 49 years-M | Piperacillin-tazobactam, Dexamethsone and Enoxaparin | EXPIRED |
| Uggeri et al [26] | P1 | 40 years-F | Hydroxychloroquine, Azithromycin,  Tocilizumab, Methylprednisolone, Morphine | RECOVERED |
| Malle et al [27] | 12 | Mean age=54 years  6-M  6-F | NA | 9 patients- RECOVERED  3 patients- EXPIRED |
| Silva et al [28] | P1 | 47 years-F | Clarithromycin, Ceftriaxone and Dexamethasone | EXPIRED |
|  | P2 | 33 years-F | Ceftriaxone, Azithromycin and Oseltamivir | EXPIRED |
|  | P3 | 29 years-M | Ceftriaxone, Azithromycin and Dexamethasone | EXPIRED |
| Abbreviation: P, patient. | | | | |
